# Monocytes isolated by positive and negative magnetic sorting techniques show different molecular characteristics and immunophenotypic behaviour

**DOI:** 10.12688/f1000research.12802.3

**Published:** 2018-03-28

**Authors:** Jashdeep Bhattacharjee, Barun Das, Alaknanda Mishra, Preeti Sahay, Pramod Upadhyay

**Affiliations:** 1 Division of Gastroenterology, Hepatology and Nutrition, Children's Hospital Los Angeles, Los Angeles, CA, USA; 2 National Institute of Immunology, New Delhi , India

**Keywords:** immune-magnetic cell sorting, lipopolysaccharide sensitivity, CD14+ve monocytes

## Abstract

**Background**: Magnetic sorting of cells, based on  microbead conjugated antibodies (Abs), employs positive as well as negative immunomagnetic separation methods, for isolation of a specific cell population. These microbeads are suggested to be nontoxic, biodegradable carriers conjugated to various antibodies. Isolation of cells through positive selection involves the attachment of antibody conjugated microbeads to the cells of interest, followed by their isolation in the presence of a strong magnetic field to obtain higher purity. Negative selection involves attachment of microbead conjugated antibodies to all other cell populations except the cells of interest, which remain untagged. In the present study, we compared the two methods for their effect on functional and immunophenotypic behavior of isolated CD14+ monocytes.

**Methods**: Peripheral blood mononuclear cells (PBMCs) were isolated from blood collected from healthy volunteers by density gradient centrifugation. Human blood derived monocytes were isolated through positive selection and negative selection, making use of the appropriate monocyte isolation kit. Monocytes were then stimulated with lipopolysaccharide (LPS) and their activation and proliferation capacity were examined. The degradation or dissociation of cell-bound microbeads was also investigated.

**Results**: We observed an impaired LPS sensitivity as well as poor activation and proliferation capacity upon stimulation by LPS in positively sorted CD14+ monocytes as compared to negatively sorted CD14+ monocytes. The attached microbeads did not degrade and remained attached to the cells even after 6 days of culture.

**Conclusions**: Our results suggest that positively sorted CD14+ cells exhibit hampered functionality and may result in inaccurate analysis and observations in downstream applications. However, these cells can be used for immediate analytical procedures.

## Introduction

Magnetic sorting is a common technique used to obtain a highly pure population of cells of interest from a mixed population of cells, making use of microbead conjugated antibodies against the cell surface antigen. Positive sorting involves the tagging of cells with magnetic microbead conjugated antibodies, followed by isolation of the labeled cells by placing them in a magnetic field. After positive sorting, cells that have microbead conjugated antibodies on their surface can be conveniently analyzed using flow cytometry ( Miltenyi *et al*., 1990; Pei *et al*., 1998). Negative sorting involves the labeling of all cells, except the cells of interest, by incubating them in a cocktail of magnetic microbead conjugated antibodies and subsequently removing them by placing them in a magnetic field.

Cluster of differentiation 14 (CD14) are specific markers used to identify monocytic populations, and they act as a coreceptors for LPS ( Guha & Mackman, 2001). Since CD14 lacks a cytoplasmatic domain, the positive magnetic sorting of CD14 may not be expected to trigger any signal transduction pathways or alter its functionality. However it has been demonstrated that anti-CD14 antibody reduces LPS responsiveness of monocytes ( Kim & Kim, 2014).

We investigated the immunophenotypic behaviour and molecular characteristics of monocytes after both positive and negative sorting, by analyzing their response and proliferation to stimuli like LPS. The biodegradation profile of the attached microbeads from the CD14+ cells was also investigated.

## Methods

### Ethical statement

The investigation was approved (project serial number: IHEC/#52/10) by the Institutional human ethics committee of the National Institute of Immunology, New Delhi-67, India.

### Isolation of PBMCs

Experiments were performed at the National Institute of Immunology, New Delhi. 20 ml of peripheral blood was collected from five healthy volunteers aged between 25–30 years, after obtaining their written informed consent. Blood was collected more than once from some of the volunteers and there was a minimum gap of three months between two successive sample collections. The peripheral blood mononuclear cells (PBMCs) were isolated from blood by density gradient centrifugation using HiSep ^TM^ LSM 1077 (Himedia, Mumbai; India). The obtained PBMCs were washed thrice with Dulbecco’s phosphate buffered saline (Himedia, Mumbai; India) and counted using the trypan blue dye exclusion method with a hemocytometer (Rohem Industries Pvt Ltd, India).

### Isolation of monocytes by magnetic activated cell sorting

Human blood derived monocytes were sorted using anti-human CD14 MicroBeads (Miltenyi Biotec, Bergisch Gladbach, Germany), as per manufacturer’s protocol. Similarly, monocytes were isolated by negative sorting using the monocyte isolation Kit II (Miltenyi Biotec, Bergisch Gladbach; Germany) according to manufacturer’s protocol.

### Cell culture and antibody dissociation assay

The positively sorted CD14 positive cells were re-suspended in RPMI 1640 medium (Himedia, Mumbai; India) supplemented with 10% fetal bovine serum (FBS) (Biological Industries, Beit-Haemek Israel) and 1X antibiotic-antimycotic solution containing streptomycin sulphate, penicillin and amphotericin-B (Himedia, Mumbai; India), and plated at a density of 4 × 10 ^6^ cells per well in 6 well low adherence plates (Corning, Tewksbury; USA). The cells were periodically harvested by gentle scrapping and passed through a magnetic column. Cells with and without bound microbeads (obtained in the flowthrough) were counted using a hemocytometer (Rohem Industries Pvt Ltd, India).

### LPS stimulation of sorted monocytes

Monocytes separated either by positive or negative selections were re-suspended in RPMI 1640 media supplemented with 10% FBS and 1X antibiotic-antimycotic solution. 1 million cells/ well were plated in a 24 well cell culture plate (Corning, Tewksbury, USA) and placed in a humidified CO _2_ incubator (ShelLab, Cornelius; USA) at 5% CO _2_/ 37°C for 24 hours. The cells were examined for adherence and thereafter stimulated with 1 ml complete RPMI media containing 500ng/ml of LPS (Sigma, St. Louis; USA). Fresh media containing 500ng/ml of LPS was replaced at each time point (8h, 16h, 24h) for supernatant collection.

### Cytometric bead array

The supernatants collected at various time points were analysed for the presence of pro- and anti-inflammatory cytokines; IL-8, IL-10, TGF-β1 and RANTES, using cytometric bead array (CBA) Soluble Protein Flex Set (BD Biosciences, San Jose; USA) as per manufacturer’s protocol. Equal volumes of five independent samples were pooled and undiluted samples were analysed. The data was recorded using BD FACSVerse (BD Biosciences, San Jose; USA) and was analysed using FCAP Array software v3.0 (BD Biosciences, San Jose; USA). The assay samples were appropriately diluted to match the detection range of the CBA kit.

### Live cell imaging

The magnetic sorted monocytes were plated at a density of 1 million cells per well in a 24 well plate. After allowing the cells to adhere for 24 hours, RPMI 1640 media supplemented with 10% FBS and 1% antibiotic-antimycotic solution containing 500 ng/ml of LPS was added to the respective wells. The culture plates were then placed in Cell-IQ (CM Technologies, Tampere; Finland) and specific fields were focussed using 10X objective magnification. Time lapse microscopy was performed and analysed for 76 hours at 30 minutes interval using live cell imaging and software (Cell IQ Analyser, Finland).

## Results

### Activation and proliferation of sorted CD14+ monocytes

**Figure 1.**
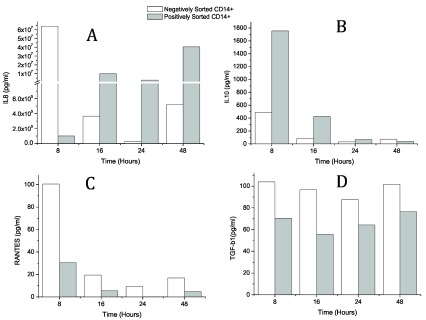
The levels of IL-8, IL-10, TGF-β1 and RANTES in 5 pooled samples at different time points after positively and negatively isolated CD14+ monocytes were stimulated with LPS.

Sorted monocytes incubated with LPS were examined for secretion of various pro- and anti-inflammatory cytokines. The levels of IL-8, IL-10, TGF-β1 and RANTES at different time points after positively and negatively isolated CD14+ monocytes were incubated with LPS were analyzed. The secretion of IL-8 was observed to be at its maximum at 8 hours in negatively sorted monocytes and at 16 hours in positively sorted monocytes ( Figure 1A). The IL-8 level in negatively sorted CD14+ cells was 6 times higher than positively sorted CD14+ cells. A similar pattern was observed for secretion of RANTES ( Figure 1C) and TGF-β1 ( Figure 1D), though the differences were not very pronounced. It was of significance to observe the reversed pattern for an anti-inflammatory cytokine, IL-10 ( Figure 1B).

**Figure 2.**
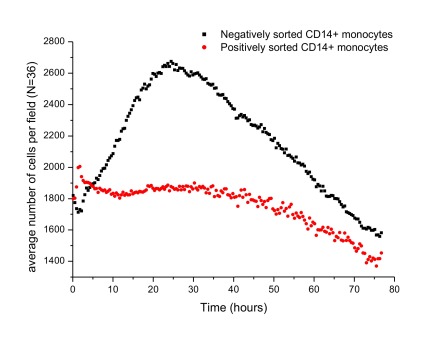
The changes in the average number of cells per field (487μmX364μm) over time, upon stimulation by LPS in positively and negatively sorted cells.

Greater secretion of the pro-inflammatory cytokines in negatively sorted CD14+ monocytes upon activation was observed, during proliferation of activated monocytes. Figure 2 shows the variation in the average number of cells per field (487μm×364μm) for positively and negatively sorted cells. Further, the progression of proliferation is shown in Video V1 for negatively sorted cells and Video V2 for positively sorted cells. Alongside Figure 2, the videos show that negatively sorted cells proliferated rapidly and extensively upon activation, and the maximum number of cells was reached after 16 hours. However, there was no clearly defined time point of maximum number of cells reached by positively sorted cells.

### Degradation of microbeads

**Figure 3.**
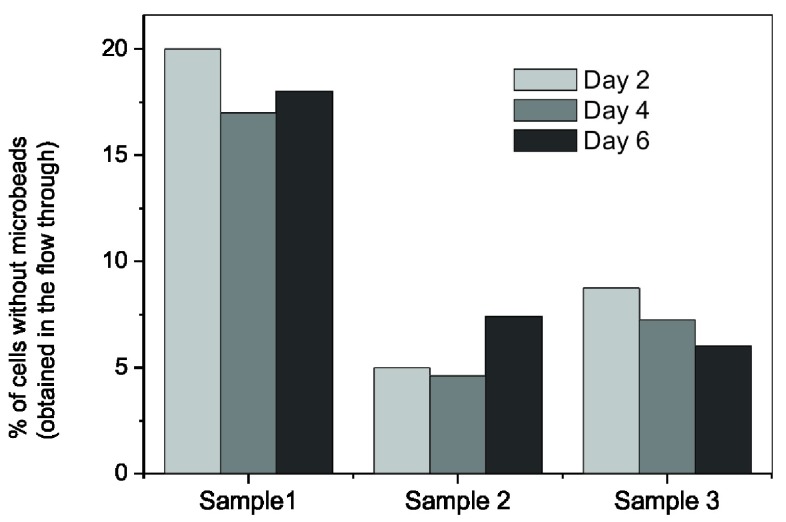
Variation in the percentages of CD14+ cells which do not have bound anti-CD14 microbeads when they were cultured for several days.

To examine the degradation of microbeads, PBMCs were labeled with anti-human CD14 antibody conjugated microbeads and sorted under a magnetic field. The sorted cells were maintained in a culture and the numbers of cells with and without microbeads were counted via flow cytometric analysis. Results of three independent samples are shown in Figure 3.

These results show the variation in percentage of cells collected in the ‘flow through’ of column placed in magnetic field. These were the cells from which the microbeads were either degraded or detached and cells were without microbeads. In all the three cultures we examined, the percentage of cells without microbeads was different but there was hardly any change in these percentages when the culture was continued for 6 days. This suggests that in typical culture conditions the Ab-microbeads remain bound with cells for many days.

Raw data corresponding to the results shown in Figure 1 Click here for additional data file. Copyright: © 2018 Bhattacharjee J et al. 2018 Data associated with the article are available under the terms of the Creative Commons Zero "No rights reserved" data waiver (CC0 1.0 Public domain dedication).

Raw data corresponding to the results shown in Figure 2 Click here for additional data file. Copyright: © 2018 Bhattacharjee J et al. 2018 Data associated with the article are available under the terms of the Creative Commons Zero "No rights reserved" data waiver (CC0 1.0 Public domain dedication).

Raw data corresponding to the results shown in Figure 3 Click here for additional data file. Copyright: © 2018 Bhattacharjee J et al. 2018 Data associated with the article are available under the terms of the Creative Commons Zero "No rights reserved" data waiver (CC0 1.0 Public domain dedication).

## Discussion

Magnetic cell sorting for the separation of large numbers of cells according to specific cell surface markers is a technique that is commonly used. It is a common notion that magnetic beads are biodegradable, do not activate cells and do not affect downstream application. We have however observed that the activation and proliferation of positively sorted CD14+ cells is impaired compared to the negatively sorted cells.

The activation of monocytes by LPS is known to occur through surface CD14, which is an LPS sensing receptor. Surface CD14 plays a crucial role; it binds and transfers LPS to the surface via TLR4:MD2 complex to enable its recognition. The LPS stimulation of monocytes activates several intracellular signaling pathways which in turn activates a variety of transcription factors ultimately leading to induction of many genes encoding inflammatory cytokines ( Guha & Mackman, 2001). In short, CD14 is involved in the LPS-induced release of IL-8, which is an important pro-inflammatory cytokine ( He *et al*., 2013).

After positively sorting CD14+ monocytes from PBMCs, the surface CD14 molecules on monocytes are blocked by anti-CD14 microbeads and these CD14+ surface sites can no longer mediate the stimulation by LPS and the positively sorted CD14+ monocytes may show impaired stimulation by LPS.

In two experiments, identical numbers of CD14+ monocytes isolated by positive and negative sorting were stimulated with LPS and their activation and proliferation was monitored. Figure 1 shows that upon stimulation by LPS the negatively sorted CD14+ monocytes secreted enormous amount of IL-8 almost instantaneously and they exhibited acute proliferation ( Figure 2). This is in accordance with the observation that IL-8 transcript is highly expressed in LPS-stimulated monocytes ( Standiford *et al*., 1992; Suzuki *et al*., 2000).

The positively sorted CD14+ monocytes responded only after 24 hours, and their level of stimulation was impaired and the cells did not proliferate. This delayed and reduced stimulation by LPS is due to the CD14 independent receptors which function to direct LPS mediated cytokine secretion under conditions where the CD14 dependent pathway is blocked or non-functional ( Lynn *et al*., 1993). There are a few LPS-associated cell surface proteins which are distinct from CD14 and these surface proteins too can bind TLRs to initiate a response ( Triantafilou *et al*., 2001). Our results suggest that the density of these surface proteins is low compared to CD14 as lesser stimulation was observed when CD14 surface groups were blocked by Abs and secondly, the delayed stimulation indicate that most likely a different pathway was followed for their activation.

Further, the secretion of somewhat higher amount of IL-10 by positively sorted CD14+ monocytes only suggest the absence of highly inflammatory conditions upon LPS activation. The levels of RANTES and TGF-β1 also indicate that the LPS activation of monocytes via CD14 independent receptors produces unique results which could be very different from common experimental situation. 

In one such related report, the, human primary monocytes were isolated by either positive or negative immunomagnetic selection and differentiated to macrophages ( Neu *et al*., 2013). The phagocytosis of *Listeria monocytogenes* (Lm) by GM-CSF-derived macrophages (GM-M) was markedly influenced by the method used for isolation of monocytes. The GM-M derived from negatively isolated monocytes showed low phagocytosis of Lm whereas GM-M generated from positively selected monocytes displayed high phagocytosis of Lm. The paper concludes that macrophages derived by *ex vivo* differentiation of negatively selected human primary monocytes as the most suitable model to study Lm infection of macrophages. In yet another report ( Elkord *et al*., 2005) it was demonstrated that the human dendritic cells generated from positively isolated monocytes by anti-CD14-coated microbeads show impaired induction by LPS.

Beliakova-Bethell *et al.* ( Beliakova-Bethell *et al*., 2014) examined the gene expression profiles of CD8+ T cells, B cells and monocytes isolated using positive selection, negative selection and FACS. They concluded that gene expression signatures were more similar between cells isolated by negative selection and FACS compared to cells isolated by positive selection. These findings were made on cells immediately after isolation and our findings establish the long-term effects of positive and negative isolation methods. In these reports it was not investigated further why the positively isolated monocytes were not suitable and had poor cytokines production upon stimulation. Our data suggests that in these experiments, the positively isolated monocytes were tagged permanently with anti-CD14 molecules attached with microbeads. The positively isolated CD14+ monocytes are identical with monocytes whose surface CD14 molecules have been blocked by Abs and these monocytes are known to behave differently ( Delirezh *et al*., 2013; Elkord *et al*., 2005; Kim & Kim, 2014).

An alternate strategy to positively isolate monocytes could be by using anti-CD33 coated beads instead of anti-CD14 coated beads. The monocytes isolated using this approach too most likely will result in impaired response upon LPS stimulation. It has been reported that CD33 and CD14 are colocalized on cell surface and when monocyte-derived immature dendritic cells were stimulated with LPS in the presence of CD33 antibody, the production of IL-12 and phosphorylation of NF-κB decreased significantly ( Ishida *et al*., 2014). The monocytes culture in the presence of anti-CD33 and the positively isolated monocytes using anti-CD33 coated beads, both have antibody bound CD33, and both are likely to respond in a similar manner.

In this context, diverse outcome could be observed when cells positively isolated by antibody bound microbeads were used for extended culture work ( Govers *et al*., 2012; Greish *et al*., 2012; Lapenna *et al*., 2013; Meinhardt *et al*., 2012).

There are two important findings from these experiments; positively isolated CD14+ monocytes have impaired LPS sensitivity and magnetic beads used in positive isolation do not degrade within days. These conclusions suggest that for most experiments, positively isolated cells are usable for analysis purpose only and should not be used for any further culture experiments.

## Data availability

The data referenced by this article are under copyright with the following copyright statement: Copyright: © 2018 Bhattacharjee J et al.

Data associated with the article are available under the terms of the Creative Commons Zero "No rights reserved" data waiver (CC0 1.0 Public domain dedication). 

Dataset 1: Raw data corresponding to the results shown in Figure 1. DOI, 10.5256/f1000research.12802.d188477 ( Bhattacharjee *et al*., 2017a)

Dataset 2: Raw data corresponding to the results shown in Figure 2. DOI, 10.5256/f1000research.12802.d182357 ( Bhattacharjee *et al*., 2017b)

Dataset 3: Raw data corresponding to the results shown in Figure 3. DOI, 10.5256/f1000research.12802.d182358 ( Bhattacharjee *et al*., 2017c)

## Supplementary material

**Supplementary Video V1:** The progression of proliferation after stimulation by LPS in negatively sorted CD14+ monocytes.

**Supplementary Video V2:** The progression of proliferation after stimulation by LPS in positively sorted CD14+ monocytes.
