# Supplementary material for: Monocytes isolated by positive and negative magnetic sorting techniques show different molecular characteristics and immunophenotypic behaviour
Source: F1000Res. 2018 Mar 28;6:2045. Originally published 2017 Nov 23. [Version 3] doi: 10.12688/f1000research.12802.3 (PMC5871943; doi:10.12688/f1000research.12802.3)
Supplement: Peer review history

# Peer review: Referee response for version 2

**DOI:** 10.5256/f1000research.14717.r31885

**Published:** 2018-03-23

**Authors:** Aniruddha Roy

**Affiliations:** 1 Department of Pharmacy, Birla Institute of Technology & Science, Pilani (BITS), Pilani, India

In the current manuscript, the authors have investigated the impact of positive selection and negative selection on the activation, function, and proliferation of monocytes. It is an important study as many researchers use these techniques interchangeably, greatly impacting the final outcome of the study and erroneous conclusion. Though the current study has used only a few experiments to draw the conclusion, their study design was neat.

 The authors may clarify some minor points to make the manuscript better:

1. In the introduction section, the authors have written: "CD14 lacks a cytoplasmatic domain and such cells are reported to function in a restricted manner" - a brief explanation of what is meant by restricted manner would be helpful.
2. The sentence on page number 5 reads: 'These were the cells from which the microbeads were either degraded or " **detected**" and cells were without microbeads.' : Probably instead of detected, it will be detached. 
3. Is the size of the beads known? Can there be a size-dependent effect due to steric hindrance of the CD14 bound beads to the TLR4? If only antibody is used, can we see the same effect? The main function of CD14 in LPS signaling in monocytes is it binds with LPS binding protein (LBP). The role of LBP appears to be that of aiding LPS to dock at the LPS receptor complex by initially binding LPS and then forming a ternary complex with CD14, thus enabling LPS to be transferred to the LPS receptor complex composed of TLR4 and MD-2. As in these in vitro studies, LBP has not been used, probably CD14 may not directly affect the LPS binding with the TLR4 receptor.

I have read this submission. I believe that I have an appropriate level of expertise to confirm that it is of an acceptable scientific standard.

# Response

**Published:** 2018-03-26

**Authors:** Pramod Upadhyay

**Affiliations:** National Institute of Immunology

Thank you for providing us valuable comments. We have addressed all of them in the revised version of the manuscript.

 Specifically

*In the introduction section, the authors have written: "CD14 lacks a cytoplasmatic domain and such cells are reported to function in a restricted manner" - a brief explanation of what is meant by restricted manner would be helpful.*

 It was an inadvertent mistake to write an incomplete sentence. This has been corrected in the revised manuscript.    

*The sentence on page number 5 reads: 'These were the cells from which the microbeads were either degraded or "detected" and cells were without microbeads.' : Probably instead of detected, it will be detached.*

 The correction has been made in the revised manuscript.

*Is the size of the beads known? Can there be a size-dependent effect due to steric hindrance of the CD14 bound beads to the TLR4? If only antibody is used, can we see the same effect? The main function of CD14 in LPS signaling in monocytes is it binds with LPS binding protein (LBP). The role of LBP appears to be that of aiding LPS to dock at the LPS receptor complex by initially binding LPS and then forming a ternary complex with CD14, thus enabling LPS to be transferred to the LPS receptor complex composed of TLR4 and MD-2. As in these in vitro studies, LBP has not been used, probably CD14 may not directly affect the LPS binding with the TLR4 receptor.*

 Nano meter size beads are used is cells isolation and these are unlikely to create significant steric hindrance. The main point is that once Ab-conjugated beads get bound to the antigen cell surface (CD14 in present case); it is bound to influence the expression, transcription and translation machinery inside the cell. And secondly, as a result of conjugation of cell surface CD14 molecule with Ab-conjugated bead in cells isolation step, the cell surface CD14 molecule would no longer be available for LBP to initiate the activation cascade.

 This has been elaborated in the Introduction section of the revised manuscript.

# Peer review: Referee response for version 1

**DOI:** 10.5256/f1000research.13872.r28324

**Published:** 2017-12-12

**Authors:** Nadejda Beliakova-Bethell

**Affiliations:** 1 VA San Diego Healthcare System, San Diego, CA, USA

This study assesses monocyte function following isolation of these cells from total peripheral blood mononuclear cells (PBMC) using positive or negative immunomagnetic selection. It addresses an important topic, because cell isolation procedure may affect cell function and skew conclusions obtained from *in vitro* studies of immune cells isolated from blood. Despite a limited assessment of functions and isolation methods, this study should have an impact on selection of monocyte isolation protocol when these cells are intended for investigation of specific responses, which this study tested. The study design is appropriate and conclusions drawn are adequately supported by the results. The paper may be improved, however, by providing more details in methods, raw data and discussion, and by careful revision of citations as detailed below:

 Methods:

 Cytometic bead array: there are several cytometric bead arrays available for human samples (e.g. “human inflammatory cytokine kit”, “human chemokine kit”). Which one was used? Legend for Figure 1 indicates that 5 samples were pooled. It would be helpful to provide details on how the pooling was done (the entire samples, equal volumes, equal proportions?) and what dilutions were made for the assay.

 Live cell imaging: It appears that information in methods contradicts the data shown in Figure 2. According to the figure and supplied raw data, imaging was performed over a course of over 76 hours, while methods section states “48 hours”.

 Raw data:

 Data for Figure 1: it might be easier to understand the data if columns were labeled by cytokine and sample (e.g. IL8 negative selection, IL8 positive selection, etc. as opposed to IL8B, IL8C, etc.)

 Data for Figure 2: were 36 fields for each sample for each time point individually counted, as the information on the figure indicates? Raw data appears to have only the averages. Was the distribution of counts tight, or was there high variation? Including raw data for individual field counts might be more appropriate.

 Discussion:

 Another option to positively isolate monocytes is to use anti-CD33 instead of anti-CD14 coated beads. This may be more appropriate in studies that aim to measure response to LPS, when positive selection is preferred (e.g. in cases of limited sample from which sequential separation of different lymphocyte subsets is desired). It might be worthy to discuss this option.

 Citations:

 Several citations throughout the paper appear to be inaccurate; for example, in Introduction some citations on altered behavior of cells following microbead attachment: (1) Safarik and Safarikova is a method review, and Horgan et al. is a protocol; neither studies cell behavior; (2) Semple et al. observed no functional changes in limited tests they did; the difference that they report pertains to CD19+ vs CD19- cells, not positively isolated vs negatively isolated cells; (3) Fuchslin et al. labeled bacterial cells for their quantification in water; and Ribaut et al. labeled parasites for their isolation from infected blood for research purposes; the relevance of these two citations for the present study may be questionable. In Discussion, Adams et al. describe a special case of MACS for multitarget sort, and may not be appropriate for referencing commonality of magnetic sorting. While the results from the present study are consistent with gene expression studies by Beliakova-Bethell et al. in terms of effects of positive selection, the reference to Beliakova-Bethell et al. is made following description of Neu et al. and Elkord et al. studies, stating that the findings were similar. This is not accurate because Neu et al. and Elkord et al. performed functional assessment following cell separation, while Beliakova-Bethell et al. lysed the cells immediately after isolation and did not measure cell function. Instead of the statement of similarity of findings, it might be worth pointing out that positive selection affects monocytes the most both in the short term and in the long term.

I have read this submission. I believe that I have an appropriate level of expertise to confirm that it is of an acceptable scientific standard.

# Response

**Published:** 2017-12-18

**Authors:** Pramod Upadhyay

**Affiliations:** National Institute of Immunology

Thank you for providing us valuable comments. We have addressed all of them in the revised version of the manuscript.

 Specifically

*Methods:*

*Cytometic bead array: there are several cytometric bead arrays available for human samples (e.g. “human inflammatory cytokine kit”, “human chemokine kit”). Which one was used? Legend for Figure 1 indicates that 5 samples were pooled. It would be helpful to provide details on how the pooling was done (the entire samples, equal volumes, equal proportions?) and what dilutions were made for the assay.*

 We used Cytometric Bead Array (CBA) Soluble Protein Flex Set and equal volumes of the samples were pooled. The CBA assay was performed on culture supernatants without any dilution. These details have been included in the revised manuscript.

*Live cell imaging: It appears that information in methods contradicts the data shown in Figure 2. According to the figure and supplied raw data, imaging was performed over a course of over 76 hours, while methods section states “48 hours”.*

 Live cell imaging: The imaging was performed till 76 hours and it was a mistake to mention this as 48 hours in the method section, we thank you for pointing out this anomaly and this has been corrected in the revised manuscript.

*Raw data:*

*Data for Figure 1: it might be easier to understand the data if columns were labeled by cytokine and sample (e.g. IL8 negative selection, IL8 positive selection, etc. as opposed to IL8B, IL8C, etc.)*

 Suggested changes have been made.

*Data for Figure 2: were 36 fields for each sample for each time point individually counted, as the information on the figure indicates? Raw data appears to have only the averages. Was the distribution of counts tight, or was there high variation? Including raw data for individual field counts might be more appropriate*.

 During the live cell imaging experiment images were logged and later cells were counted using the Cell-IQ Analyser software. The protocol for cell counting in the analysis software was assembled to finally provide the average number of cell count in the selected fields. The cell counts from individual fields of various images were embedded in the analysis software, thought it was possible to export these tables but due to very large number of data files we focussed only on the averages. 

*Discussion:*

*Another option to positively isolate monocytes is to use anti-CD33 instead of anti-CD14 coated beads. This may be more appropriate in studies that aim to measure response to LPS, when positive selection is preferred (e.g. in cases of limited sample from which sequential separation of different lymphocyte subsets is desired). It might be worthy to discuss this option.*

 This is a good suggestion keeping in mind that the CD14 is an endogenous ligand for CD33 in monocyte-derived immature dendritic cells. It has been reported (Hiroshi Nakada et al.; J Biol Chem. 2014 Sep 5; 289(36): 25341–25350) that when monocyte-derived immature dendritic cells were stimulated with LPS in the presence of anti-CD33Ab, the production of IL-12 and phosphorylation of NF-κB decreased significantly.

 Most likely the positively isolated monocytes using CD33-Ab beads would behave in a similar manner.

 This possibility has been discussed in the Discussion section of the revised manuscript.

*Citations:*

*Several citations throughout the paper appear to be inaccurate; for example, in Introduction some citations on altered behavior of cells following microbead attachment:*

 We once again thank the reviewer for thoroughly examining the citations; the list of references has been appropriately revised.

 The differences between the findings of  Beliakova-Bethell et al. and Neu et al. & Elkord et al. have been incorporated in the revised manuscript.
